# *SmilesUp* text message intervention for early childhood dental caries prevention: A protocol for a randomised controlled trial

**DOI:** 10.1371/journal.pone.0310561

**Published:** 2024-09-30

**Authors:** Rebecca Chen, Michelle Irving, Bradley Christian, Neeta Prabhu, Harleen Kumar, Woosung Sohn, Heiko Spallek, Simone Marschner, Clara K. Chow

**Affiliations:** 1 The University of Sydney, Westmead Applied Research Centre, Westmead, NSW, Australia; 2 The University of Sydney, School of Dentistry, Camperdown, NSW, Australia; 3 Oral Health Services, Western Sydney Local Health District, Westmead, NSW, Australia; 4 Sydney Dental Hospital and Oral Health Services, Sydney Local Health District, Sydney, NSW, Australia; University of Maribor, SLOVENIA

## Abstract

**Introduction:**

Despite improvements in health outcomes for children, early childhood dental caries (ECC) remains a chronic lifestyle-mediated disease that affects an estimated 600 million children worldwide. Parental influence on oral health-promoting behaviours is pivotal in preventing ECC. The latest WHO publications *Ending Early Childhood Dental Caries* and *Mobile Technologies for Oral Health* highlights the opportunity for mobile Health programs (including text message programs) to improve oral health behaviours and oral health self-efficacy. In response, our team of parents, oral health professionals (academics and health promotion experts), and IT specialists co-designed a 12-week, behavioural theory-informed text message program (called *SmilesUp*) to address behavioural risk factors specific to ECC. This randomised trial aims to assess whether the *SmilesUp* program improves parents’ oral health promoting behaviours (like tooth brushing twice a day with toothpaste) and oral health self-efficacy for their children compared to usual care.

**Methods and analysis:**

A randomised controlled trial with a 1:1 parallel design will be conducted among 150 parents with children diagnosed with ECC and accessing public dental care in NSW, Australia. Patients will be stratified by hospital site, and modality of care (Dental General Anaesthetic (DGA) or not) and then randomly assigned to either immediately receive the SMILESup text messaging intervention or receive the program at the end of the study period. The primary outcome at 12 weeks is twice daily brushing with fluoride toothpaste. Secondary outcomes include changes in the intake frequency of sugared drinks and foods, oral health promoting bedtime routines and oral health self-efficacy. The primary analysis will follow an intention-to-treat principle. In addition, a process evaluation will examine barriers, enablers, and opportunities to scale the program.

**Ethics and dissemination:**

Ethics approval has been obtained from the Western Sydney Local Health District Human Research Ethics Committee 2022/ETH01920. Study results will be disseminated via peer-reviewed publications and presentations at conferences.

**Trial registration:**

**Trial registration number**: This clinical trial has been prospectively registered on the ANZCTR from the 27^th^ of March 2023. Registration number: ACTRN12623000325606.

## Introduction

Early childhood caries (ECC) remains a complex childhood problem that affects more than 600 million children worldwide [1]. It is defined as “the presence of one or more decayed (non-cavitated or cavitated lesions), missing (due to caries), or filled tooth surfaces in any primary tooth in a child under the age of six” [2]. Although dental caries is preventable, young children continue to require invasive procedures provided under dental general anaesthetics (DGA) to treat ECC [3–5]. In Australia, and New South Wales (NSW), these admissions affect one in every 250 children with a repeat admission rate of 9% within two years and a further 9.8% admission rate for subsequent children in the same family [6]. The repeat admissions also present a significant cost to individual families and the health care system [3]. Developing innovative programs that enhance parents’ daily oral health practices for their child is crucial for preventing the progression of ECC and reduce both disease recurrence and related hospital admissions [7–9]. As a non-communicable lifestyle-associated disease ECC is preventable through good oral hygiene, tooth brushing with toothpaste, diets low in free sugars and oral health promoting bedtime routines that omit milk feeding throughout the night [1,10]. The latest American Academy of Paediatric Dentistry’s policy on ECC prevention highlights the need to reduce sugar-containing foods and drinks and adopt early oral hygiene measures including twice daily brushing [11]. A recent prospective study has also shown that for children two and three of age, brushing less than twice a day would double the likelihood of future caries incidence at age five [12]. The World Health Organisation’s (WHO) latest report on *Ending Early Childhood Dental Caries* highlights the crucial role of parental knowledge, motivation and capability (i.e. oral health self-efficacy) for oral health promoting behaviours on the dental outcomes of their children [9].

New approaches for ECC disease management must consider the strategic directions provided by the WHO’s Global Health Action Plan (2023–2030) and consider the use of mobile technologies [7,13]. The WHO’s *Mobile technologies for oral health* guideline highlights using mobile health interventions as a scalable and impactful “best-buy” intervention to increase oral health literacy and improve oral health behaviours [7,9]. It defines mobile Oral Health (mOralHealth) as the use of mobile and wireless technologies (such as mobile phones and text messaging programs) to support the achievement of oral health objectives and highlights its use to complement the provision of basic clinical oral health care [14]. When applied to preventing ECC, mHealth programs can complement clinical care by providing parents and carers (referred to as parents in this paper) with additional knowledge, and positive reinforcements through messages with links to video demonstrations accessed in the home contexts where oral health habits are being formed. This type of intervention is synergistic with the WHO’s definition of health literacy which recognises the broader cognitive and social skills that determine the motivation and ability of individuals to gain access to, and understand information in ways that promote good health [15,16]. Therefore, the outcome measures for this study, although primarily behavioural will also include measures for the construct of oral health self-efficacy.

Text messaging programs, co-designed with consumers and health professionals are an effective, equitable and cost-effective solution to support behaviour change and prevent a range of non-communicable diseases [17–19]. Most emerging mHealth research addressing oral health, targets behaviour change in high-risk populations [20] by focusing a single behaviour, whether it be on oral hygiene behaviours [21], night-time routines [22] or diet [23]. A comprehensive program to address a range of lifestyle risk behaviours specific to the prevention and management of ECC has not been created in the Australian context. Specifically, a recent Australian review identified a gap and a need to co-design mHealth programs specific for parents of children 0–6 years old who have ECC [24].

In response, our team has worked with parents and a range of health professionals to co-design a text messaging program (*SmilesUp*) to support ECC prevention in the NSW public dental service context. *SmilesUp* contains 36 positively framed oral health messages assessed to be useful, readable and acceptable by consumers accessing NSW public dental services [25]. This study aimed to test the effectiveness of the *SmilesUp* intervention on oral health behavioural outcomes and self-efficacy.

## Materials and methods

### Study aim

This study aimed to test the effectiveness of the *SmilesUp* text message program to change oral health behaviours and self-efficacy of parents in a single-blinded randomised controlled trial, compared to usual care. We will also conduct a process evaluation to assess barriers and enablers of the program to inform future scale-up plans.

### Study design

**Fig 1:**
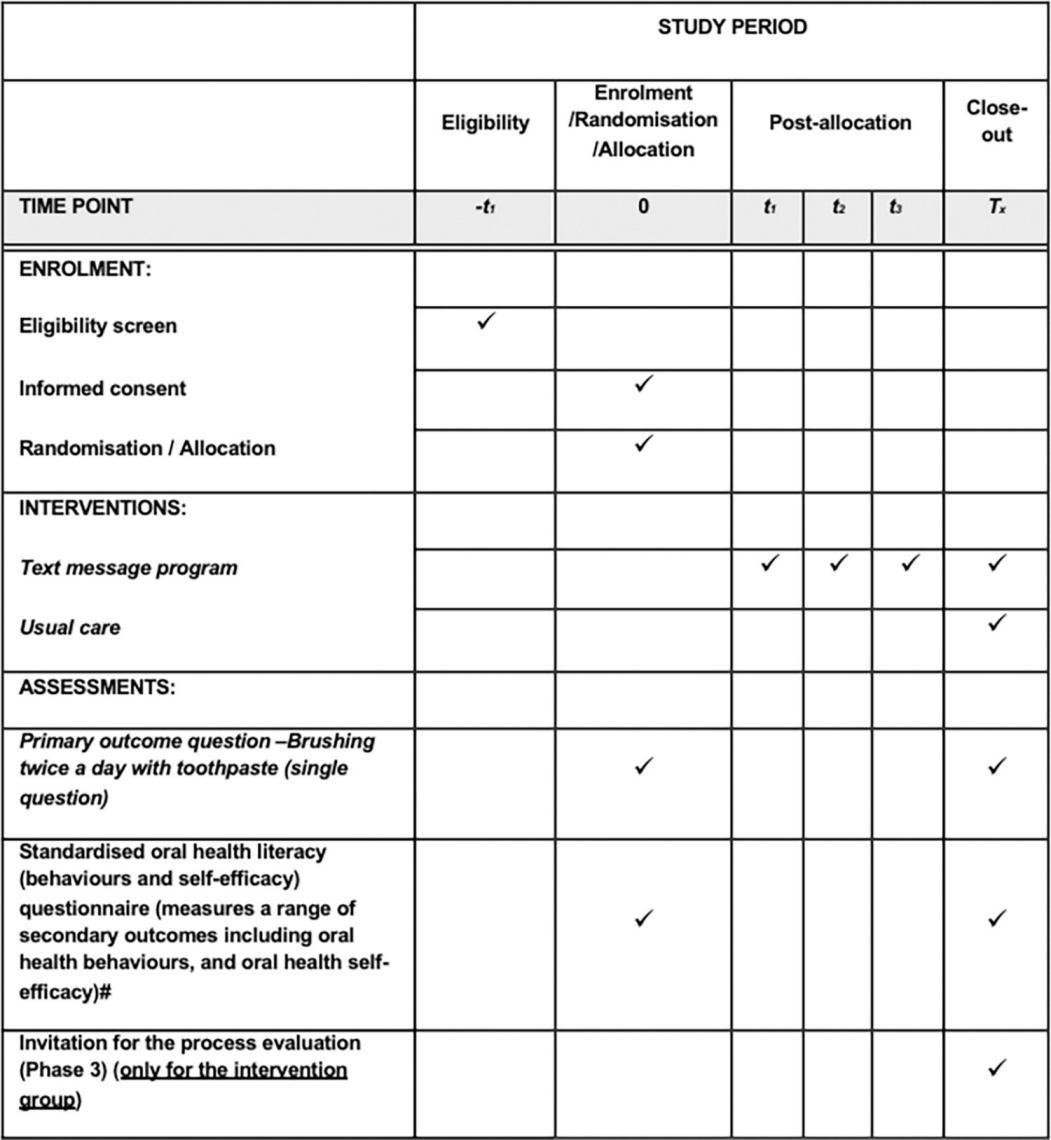
SPIRIT Schedule of enrolment, intervention, and assessments.

**Fig 2:**
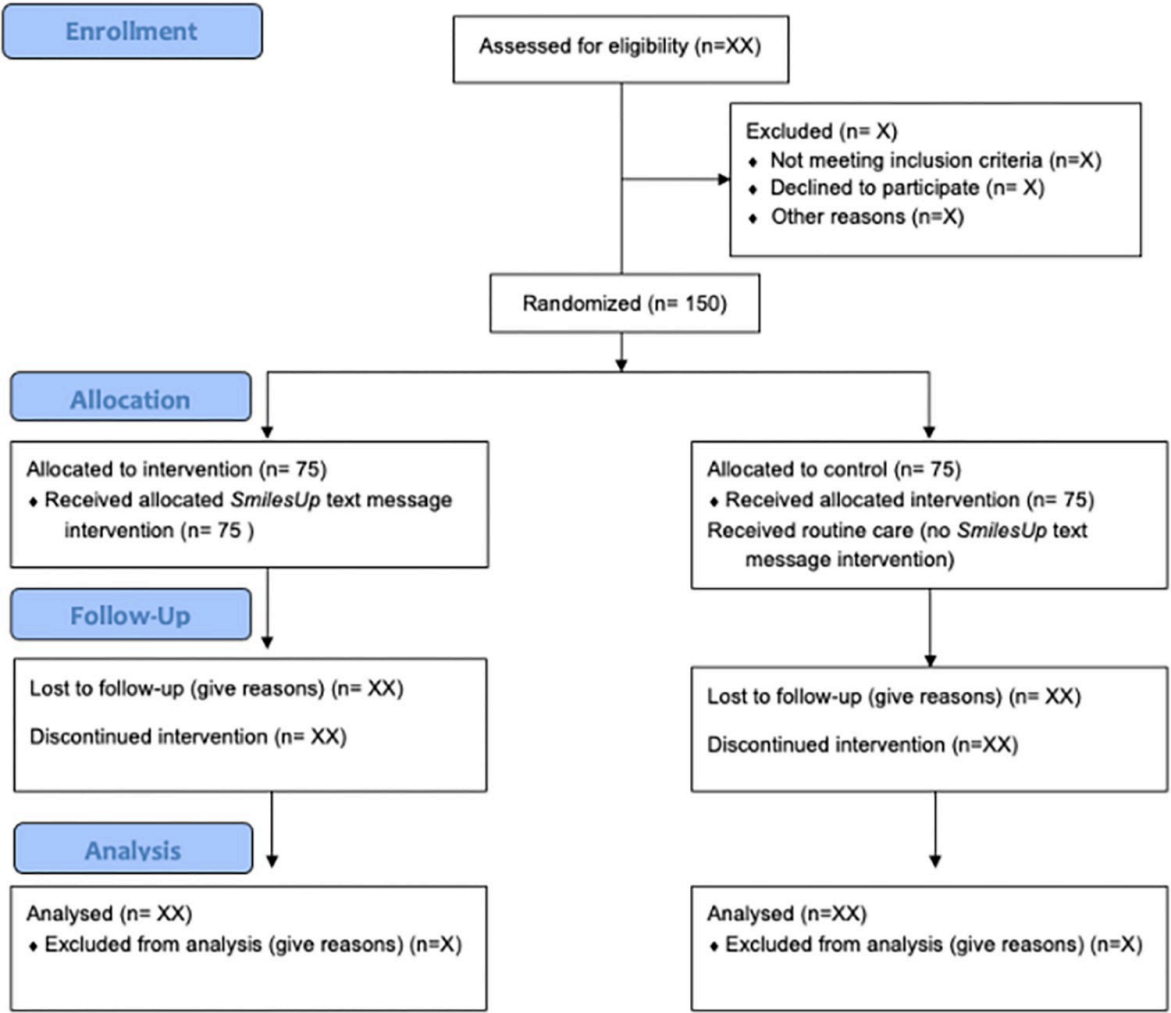
SMILESup CONSORT RCT study flow chart.

The SMILESup study is a multi-site, single-blinded (dental practitioner blinded, patient unblinded), parallel designed randomised controlled trial of 150 parents (who have children 0–6 years old inclusive with a ECC diagnosis), testing the effectiveness of a 12-week co-designed text message program (*SmilesUp*) to support oral health behaviour change in their children and parental self-efficacy compared to usual care. The intervention participants (n = 75) will receive the program after randomisation and the control group (n = 75) will receive the program after the 12-week trial period. Fig 1 shows our SPIRIT schedule of enrolment, interventions, and assessments. Fig 2 shows the CONsolidated Standards of Reporting Trials (CONSORT) RCT study flow.

### Setting

The study population will consist of parents with children diagnosed with ECC who are either awaiting or receiving dental treatment, including but not limited to DGA within the NSW public dental service, in Australia. This intervention will run alongside and be an adjunctive support tool for parents whilst their children are in a clinical course of care within the public dental service system. Children in both groups will receive appropriate clinical and preventive care, governed by the existing policies within NSW public dental services [26,27]. Specifically, this trial will take place in six local health districts across metropolitan, rural and regional areas. These community dental clinics all refer to the two tertiary paediatric dental departments within NSW Health. These clinics predominantly provide a fee-free safety-net dental service for families who cannot afford private dental care.

### Patient population

Previous studies in Australia have indicated that this population group accessing public dental care has the highest severity of disease [6] and lower uptake of oral health promoting behaviours and low oral health literacy levels [28]. A recent study showed that only 53% of this population performed the recommended oral health behaviours (tooth brushing twice a day with toothpaste) to prevent ECC for their children [29].

### Sample size

A sample size of 150 will enable the detection of a 25% difference between intervention and control groups with an 80% power. The clinically meaningful impact of the intervention was chosen with a health equity lens. A recent cross-sectional study of a similar Australian population group from lower socioeconomic areas accessing public dental services reported that 53% of children between 3–8 years old brush twice a day with toothpaste [29]. However, 78% of children in Australia from high-income families brush twice a day with toothpaste [30]. Thus, to determine a clinically meaningful difference we aimed to increase the number of families brushing twice a day with toothpaste from 53% of the population to be comparable to the 78% of children from high-income households who were achieving the target behaviour. This required an increase of 25%. Therefore, a sample size of approximately 150 will achieve an 80% power, to observe a meaningful difference of a 25% increase of participants who brush twice a day with toothpaste in the intervention compared to the control group at three months (53% to 78%), using a 5% level of significance and accounting for a 25% drop-out rate.

### Participant eligibility

Inclusion criteria are parents of children who have all the following:

1. diagnosis of dental caries (tooth decay)
2. age 0 to 6 years inclusive
3. waiting (or have attended) a public dental service for dental caries (tooth decay) within the public health system in NSW.

Parents will be excluded if they:

1. are already participating in a text message-based study on lifestyle risk factors for health.
2. have insufficient English skills to provide informed consent or read and understand text messages
3. are unable to comply with study requirements.

Parents of these children must also own an operational mobile phone capable of sending and receiving SMS text messages; and provide written informed consent.

### Recruitment and consent

Recruitment will be performed around existing clinical workflows within clinical settings in NSW public dental services. Researchers will screen parents for eligibility. Only parents of children who fit the eligibility criteria will be invited to take part in the study. These parents will be provided with a recruitment information flyer. The recruitment information flyer will contain a QR code to a Research Electronic Data Capture (REDCap) link with an Expression of Interest form and a further link to the Participant Information Consent Forms (PICF). The PICF visible via the REDCap link will contain an eConsent form that will also be held and stored securely on REDCap. Parents will have access to the PICF and be able to discuss involvement with the rest of their families and contact researchers with further questions before providing consent. Parents will also be able to take the flyer home before deciding to enrol, and the contact details of the researcher will be available in case any potential participant has further questions to enable informed consent. For consent to be obtained, the parent must complete and sign the eConsent before any data collection or intervention delivery occurs. At the parents request, paper versions of the PICF and enrolment forms will be available. The lead researcher may delegate and train available clinicians/researchers at the study sites to explain the study and answer more questions in detail.

### Randomisation and blinding

Randomisation will also occur after consent and baseline data collection. Participants will be randomly assigned to either the control or the intervention group with a 1:1 allocation ratio, stratified by recruitment site (community dental clinics vs. tertiary paediatric settings) and modality of care (DGA vs. non-DGA). Randomisation will be generated by the study statistician using the Randomise R library of R statistical software (V.4.3.2) and is based on both permuted block sizes and stratification, where stratification occurs by site (community dental clinics vs. tertiary paediatric settings) and the modality of care (DGA vs. non-DGA) only. The intervention allocation sequence will be incorporated into REDCap so that researchers recruiting and analysing the data will be blinded to treatment allocation. Participants will be notified of their treatment allocation via text message the following Monday after enrolment, thus participants will not be blinded to allocation. As the intervention is low risk and participants can opt-out, it is anticipated that emergency unblinding will not be necessary for this study.

### Intervention—The *SmilesUp* 12-week text messaging program

The *SmilesUp* text message program will deliver 36 messages over 12 weeks using a pre-programmed delivery algorithm informed by consumer engagement. We have used a co-design process that has been previously published [31], driven by behavioural theory to be synergistic with the principles in the current WHO guidelines [17,32]. These principles highlight the importance of co-designing and adapting mHealth programs for specific local contexts and target populations. By co-designing the program, we first identified that parents were seeking reminders, reassurance, and video resources from health professionals that they could access on their smartphones at home to complement the clinical care and advice they had previously received. Consumers and clinicians were also consulted about the length of the program and provided with evidence for the time taken for effective behaviour change [31,33,34]. The final pre-programmed delivery algorithm, developed with consumer input is hosted on the TextCare™ platform. The TextCare™ platform is a proprietary and custom-built platform that has been successfully used to address lifestyle behaviour change for the management of a range of cardiometabolic diseases [35,36]. The program is semi-personalised to include their child’s name in some messages and allows the parents to choose when they receive the messages based on the information provided by parents when they sign up for the study.

### Intervention development, patient, and public involvement

Intervention development occurred in a separate phase of the project before the commencement of the RCT [31]. It involved consultation at the inception of the program, with end users, namely parents accessing NSW public dental services. Our intervention development was uniquely co-designed with parent consumers (n = 20) and a wide range of health professionals (n = 17) including paediatric specialists, general dental practitioners, health promotion, health policy professionals, IT application specialists, researchers, oral health dieticians and academics working within the NSW public dental services. This process is described in more detail in a previously published conference paper [31].

A summary of the three main steps for our patient and public involvement, during the co-design process are as follows. Firstly, the parent groups were consulted to identify target behaviours barriers and enablers related to oral health promoting behaviours related to ECC.

The primary target behaviours identified included:

1. Brushing twice daily with toothpaste

Other secondary target behaviours included:

1. Reducing the consumption of sugar-sweetened beverages
2. Reducing the frequency of a child going to bed with milk in a bottle.
3. Reducing the consumption of sugary foods
4. Increasing the regular consumption of water

**Fig 3:**
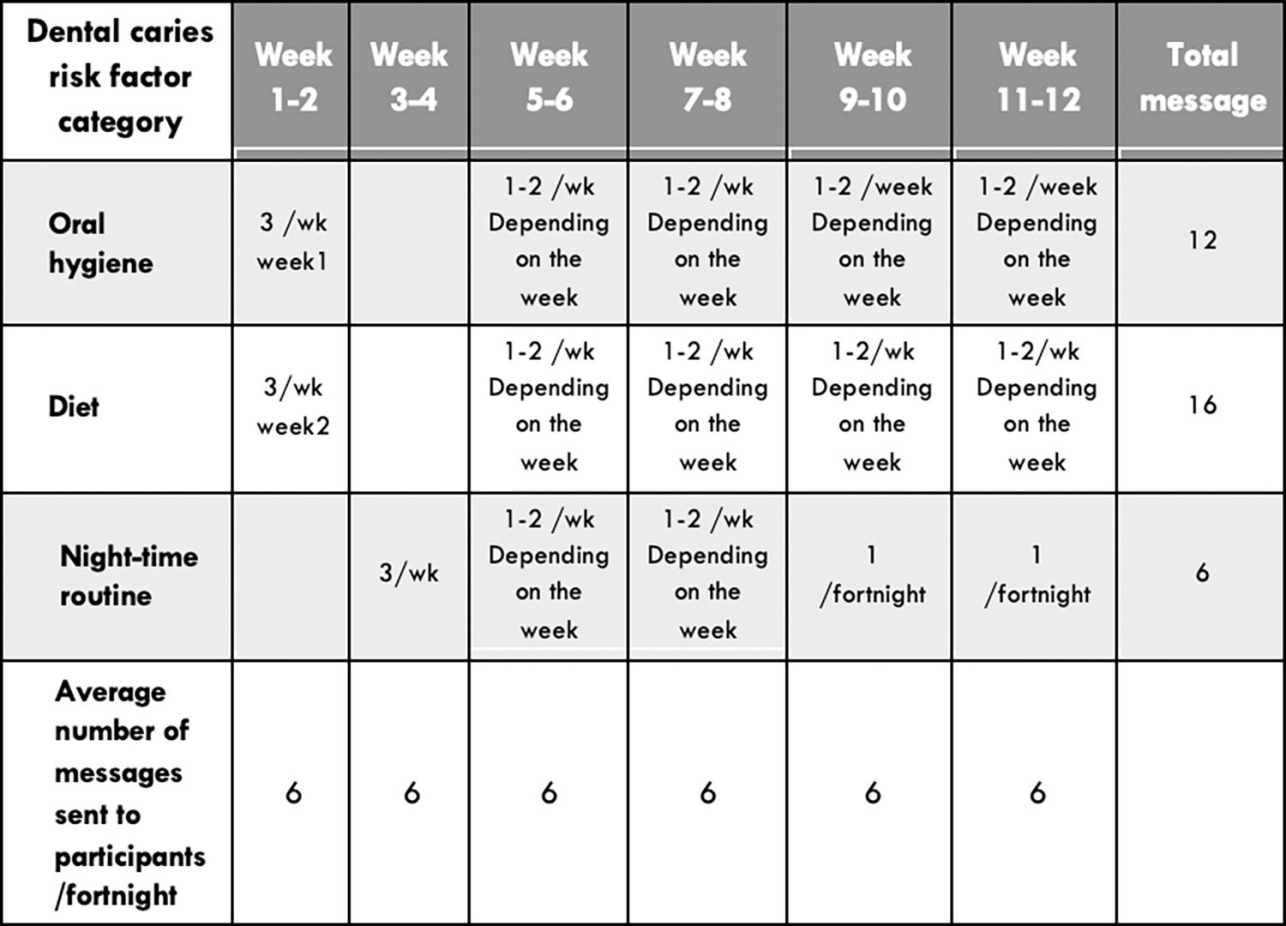
Intervention component and sequence.

**Fig 4:**
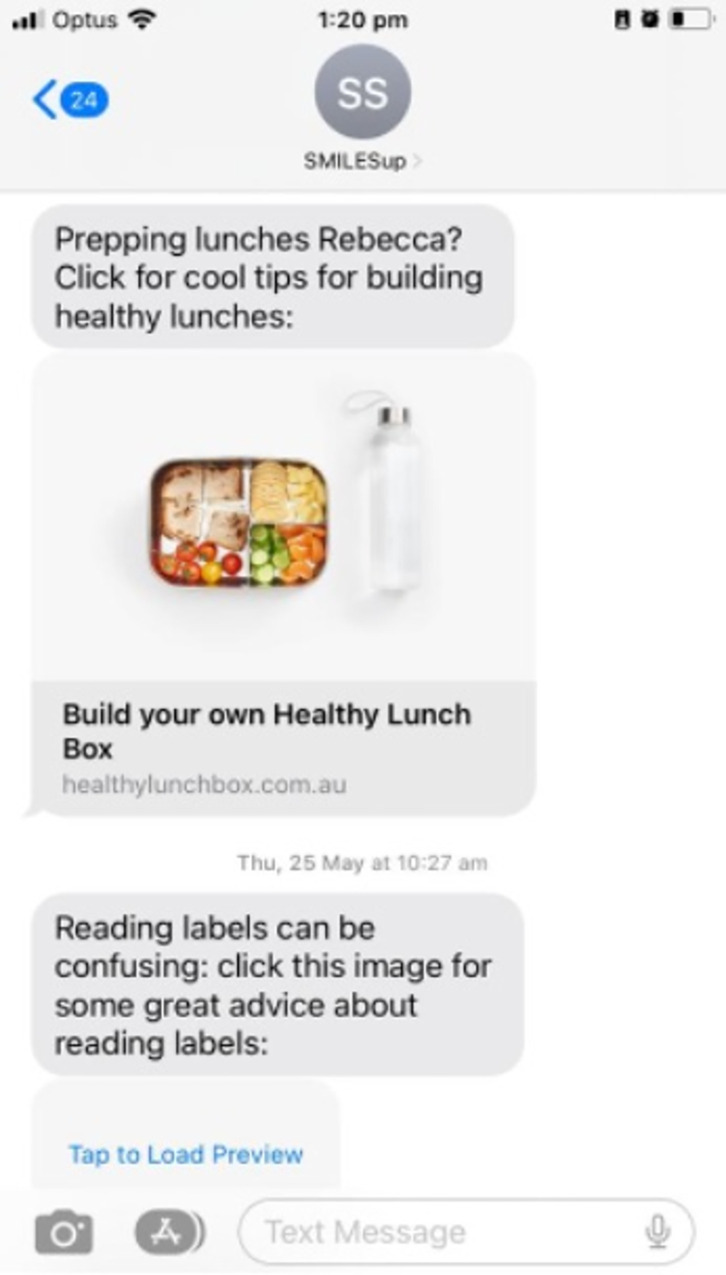
Examples of text messages.

Secondly, the group agreed to the program aims developed using the WHO mOral Health Literacy framework shown in Table 1. Additionally, interested parents and health professionals had the opportunity to draft text message content. Thirdly, a broader group of consumers and health professionals working with the target group assessed the acceptability and usefulness of the draft messages [25]. Only messages that had high acceptability and usefulness by parents and health professionals were included in the final set. The high-level sequence of the final 36 messages and examples of some messages are shown in Figs 3 and 4. Each of the final messages was mapped to the target behaviours, barriers and enablers identified by the parents and linked to a specific behaviour change techniques [31].

**Table 1:** SMILESup program aims to use the WHO moral health literacy module framework.

**Aim**	To improve the oral health behaviours and oral health self-efficacy of parents and their children with ECC.
**Implementers**	Partnership between dental practitioner and specialist within local health districts, public health professionals and academics.
**Target audience/groups**	Parents are intermediaries to reach their children aged 0–6 years old who are waiting for a dental treatment including dental care with DGA.
**Potential technologies**	Semi-personalised SMS, other media assets including URL links to video resources provided by text message.
**Primary outcomes**	Primary outcome health behaviour: Brushing twice a day with toothpaste–increase of 25% from baseline brushing levels.
**Secondary outcomes**	Secondary outcome 1) Oral health behaviours: • Use of fluoride toothpaste • Sugary food and drink consumption • Oral health promoting night-time routines • Water consumption 2) Oral health self-efficacy
**Impacts**	Short-term: effective oral health habit formation to ensure the sustainability of prevention and the self-management of ECC. Longer-term clinical outcomes: reduced decay and the need to have a repeat general anaesthetic appointment for ECC.

### Intervention group

The intervention group will receive the *SmilesUp* text message support program in addition to the usual clinical care and advice provided in the clinical setting. Participants will be informed that this is primarily a one-way messaging service and will receive their semi-personalised messages at the specific time of the day they have chosen at the beginning of the intervention.

At the end of the 12-week program, parents will be asked to fill in a standardised questionnaire to measure the change in oral health behaviours and oral health self-efficacy. On completion, parents will also have the option to attend focus groups or semi-structured interviews to provide more in-depth feedback.

### Control group

The control group will receive usual care, which consists of oral hygiene instruction and dietary advice provided in the clinical setting. The control group will enrol in the *SMILESup* study which includes completing a standardised questionnaire that records general demographic questions, baseline oral health behaviours, and self-efficacy. At the end of the 12 weeks, they will be required to complete a similar standardised post-survey questionnaire about their oral health behaviours, and self-efficacy. Upon completion of the post-survey, the control group parents will be offered the *SmilesUp* text message program.

### Data collection & study outcome measures

Data will be collected from participants at baseline and follow-up (3 months post-allocation). We developed a self-reported questionnaire to measure all behavioural and self-efficacy outcomes pre- and post-intervention available in our protocol. A summary of our outcomes is shown in Table 2 and Fig 1. Data collection for baseline and post-intervention will primarily be collected electronically using REDCap. However, parents also have the option of providing their details so that the research team can contact them or provide them with the opportunity to complete the enrolment process or post-intervention data collection at home in their own time. Where requested, hard copies of these questionnaires will also be made available.

**Table 2:** Study outcomes.

Outcome	Assessment measure
*Primary outcome*:	
**Tooth brushing for their child twice a day with toothpaste**	Self-reported questionnaire adapted from existing questions used to evaluate other NSW Health programs [37].
*Secondary outcomes*:	
**Fluoride toothpaste use**	Self-reported questionnaire adapted from existing questions used to evaluate other NSW Health programs [37].
**Sugary drinks consumption to Australian guidelines**	Self-reported questionnaire from cut-offs determined by current Australian guidelines [38] and adapted from existing questions used to evaluate other NSW Health programs [37].
**Sugary foods consumption to WHO guidelines**	Self-reported questionnaire from recommendations by the WHO guidelines on the consumption of sugars for the prevention of ECC [39] and adapted from existing questions used to evaluate other NSW Health programs [37].
**Water consumption**	Self-reported questionnaire adapted from existing questions used to evaluate other NSW Health programs [37].
**Bedtime routines (milk before or during sleep)**	Self-reported questionnaire adapted from existing questions used to evaluate other NSW Health programs [37].
**Oral health self-efficacy**	Self-reported questionnaire adapted from previous literature using the COM-B framework to measure Oral Health self-efficacy [40].

### Primary study outcome: Tooth brushing

The primary outcome is based on whether the parents adhered to the current recommended guideline and brushed their child’s teeth twice a day with toothpaste [11]. This item will be analysed as a binary (*yes/no*) response to the question adapted from an existing question used to evaluate other NSW Health programs [37] “How often do you brush your child’s teeth with toothpaste?” A *yes* response will be considered for patients answering, “Twice a day” or “More than twice a day” and a *no* response to those answering, “Once a day”, “few times a week”, or “Don’t know /unsure”.

### Secondary outcomes

Secondary behavioural outcomes will be analysed as a binary (*yes/no*) response to behavioural questions that have been adapted from existing questions used to evaluate other NSW Health programs [37]. These include:

- *Type of toothpaste used*. This secondary behavioural outcome considers whether a parent is using fluoride toothpaste according to current guidelines [9]. This will be calculated based on the response to the question: “What type of toothpaste do you put on our child’s toothbrush?” [37]. A *yes* response will be considered for “Children’s fluoride toothpaste”, “Standard fluoride toothpaste” or, “Prescribed toothpaste” and a *no* response to those answering, “Non-fluoride toothpaste” and “Don’t know/unsure.”
- *Frequency of sugared beverage consumption for the child*. This secondary behavioural outcome considers the number of sweet beverages a child has per week based on cut-offs determined by current Australian guidelines [38]. This will be calculated based on the response to the question “How often does your child drink sugar-sweetened drinks (such as soft drinks, cordials, sports drinks, flavoured milk, fruit juices, energy drinks or iced teas?)” A *yes* response will be considered for “Rarely” or “Never” and a *no* response for those answering, “Every day” “A few times a week” and “Don’t know/unsure.”
- *Frequency of sweet food consumption for the child*. This secondary behavioural outcome considers the dietary behaviours of the child related to the frequency of sweet foods per week based on recommendations from the WHO guidelines for the consumption of sugar [39]. This will be calculated based on the response to the question “How often does your child eat sweet foods like chocolates, sweetened cereal, ice cream, flavoured milk or yoghurt, biscuits, cakes, pastries, or snack/muesli bars?” A *yes* response will be considered for “Rarely” or “Never” and a *no* response for those answering, “Every day” “A few times a week” and “Don’t know/unsure.”
- *Bedtime routines related to the frequency and timing of milk consumption at night*. This secondary behavioural outcome measure considers the specific risk behaviour of consuming milk before or during sleeping hours as outlined in the WHO’s ECC report [9]. This will be calculated based on the response to the question “How often does your child go to bed with milk in a sippy cup or bottle?” A *yes* response will be considered for “Rarely” or “Never” and a *no* response for those answering, “Every day” “A few times a week” and “Don’t know/unsure.”
- *Frequency of water consumption*. This secondary behavioural outcome measures the positive behaviour of frequently consuming water. This will be calculated based on the response to the question “How often does your child drink water?” A *yes* response will be considered for “Every day” and a *no* response for those answering, “A few times a week” “Don’t know/unsure” “Rarely” or “Never.”
- *Oral Health self-efficacy*. To provide some information on the construct of oral health self-efficacy, we will collect a standardised set of six questions on each of the three target behaviours (oral hygiene, diet and bedtime routines) that were adapted from previously published literature [40]. The six questions consider capability (physical capability and psychological i.e. knowledge capability), opportunity (physical opportunity, social opportunity), and motivation (reflective motivation, and automatic motivation) [40,41]. These questions draw upon the COM-B framework which itself draws upon the Health Beliefs Model (HBM) and the Theory of Planned Behaviour (TPB) [42,43]. The total oral health self-efficacy measure for each target behaviour (oral hygiene, diet and bedtime routines) will be analysed by a composite measure out of 60. Secondary analysis for each question includes a binary measure which will be dichotomously categorised where a score of 8 or more on a 10-point Likert scale is considered a *yes* and a score below 8 is considered *no*.

### Statistical analysis

Our statistical analysis will follow an intention-to-treat principle. A full pre-specified statistical analysis plan will be developed and finalised before study completion, data lock and analysis. Normally distributed continuous variables will be expressed as means with standard deviations. Non-normally distributed variables will be expressed as medians and interquartile ranges. Binary variables will be presented as proportions with their 95% confidence intervals (CI). The proportion of parents who meet the primary outcome of toothbrushing twice a day with toothpaste at baseline and 3 months will be presented descriptively by treatment groups. An odds ratio of brushing their child’s teeth twice a day with toothpaste at 3 months comparing treatment vs. control (reference group) will be estimated with a 95% CI using a logistic regression model adjusting for whether parents are toothbrushing twice a day with toothpaste at baseline and stratification variables. All dichotomous secondary outcomes will be analysed using the same logistic regression model and continuous secondary outcomes will be analysed using linear regression. A statistical significance level of 0.05 will be used. Interaction between treatment and pre-specified covariates will be conducted to explore the heterogeneity of our results. Sub-group analyses will be conducted for treatment modality (DGA vs. non-DGA), and site (tertiary vs. non-tertiary). No multiplicity adjustments will be performed for secondary outcomes as they are exploratory by nature. We will not be conducting further interim analyses with stopping guidelines. All analysis will be undertaken using R.

### Data management plan

All data will be collected at baseline and at a 12-week follow-up (Fig 1). An enrolment log will be maintained by a delegated member of the research team with the express permission of the coordinating principal investigator (CPI). This log will be stored on LHD servers. Access to the LHD server is restricted by password, those with access must also be employees/contingency workers of that LHD. Only the CPI and delegated researchers will have access to the complete and final data set from all sites, and only available on REDCap.

Baseline data will include demographic information, baseline oral health behaviour, and self-efficacy delivered through the REDCap platform [44]. The REDCap system is an online secure capture data collection and management tool for research use. Our study will use REDCap to collect baseline and post-intervention data. REDCap has been provided by the Office for Health and Medical Research for the use of researchers in NSW Public Health LHDs, networks, and pillars. REDCap is hosted in the eHealth AWS cloud, maintained by eHealth, and supported by the Office for Health and Medical Research. It is secured, and backed up daily, where privacy and confidentiality considerations of participants are protected.

Whilst most questionnaires and PICF forms are provided electronically, there may be a small number of participants requesting hard copies. Once the hard copies are obtained, study staff at each site will log in to the REDCap database with their unique username and password to enter data. Staff will only have access to data collected at their specific site. Only coded data will be analysed. Only the chief investigator and principal investigator will have access to all study data. Hard copies of consent forms and questionnaires will be stored in lockable filing cabinets in a restricted access room, only accessible by project staff, and restricted by swipe access provided by the sites.

All data for this study will be collected from the individuals themselves. No additional data will be gathered from medical/ health records. Following the NHMRC guidelines on the management of data and information in research, paper and electronic study information, including the consent forms and questionnaires, will be securely stored until 5 years after the completion of the study. Electronic data will be permanently deleted from servers and hard copy documentation shredded and disposed of in secure confidential bins provided by hospital sites. The destruction of the data will follow processes following the University of Sydney Research Data Management processes in alignment with the NHMRC guidelines.

For the process evaluation, where Zoom or online telephone links are sent, meetings will be password protected. Further confirmation of the receipt of the PICF will be provided on the recordings. All data from the recordings will be stored on a secure encrypted server of the University of Sydney’s Research Data Store and deleted from any device. Any quantitative metrics to assess fidelity will be analysed by R [45] or SPSS v22. All qualitative data will be analysed using NVivo version 12.0 [46] coded data will be analysed in themes qualitative methodologies, following a framework analysis approach [47].

### Safety considerations

Whilst it is unlikely that there will be adverse effects from the text message intervention, our protocol ensures participants’ safety by providing a relevant contact person including a senior clinician-researcher at each of the sites and a contact person at the patient experience units to address complaints related to the study. Parent participants can opt out of the program at any time. Parents can do this by texting back “STOP”. Once they do this the participants will no longer receive messages from the text messaging platform. However, to withdraw from the study they will need to contact the principal investigator at their site to complete the withdrawal form.

The TextCare™ platform will be centrally monitored by a clinician-researcher. This centrally monitored system enables adherence to the intervention’s pre-set delivery algorithm and data collection sequence set out in this protocol. If any participant requires clinical assistance for their child’s oral health during the study period, they will be provided contact details to their nearest NSW public dental clinic. Additionally, if the patient’s condition worsens from an acute dental abscess leading to a facial swelling, they will be provided the contact details for their nearest paediatric emergency service. After the patient contacts the public dental service, an appointment will be provided according to the current eligibility policies in place within NSW public dental services [27]. The details of a senior clinician researcher for the specific sites will also be provided on the PICF. A log of the participants who have stopped the intervention, withdrawn, or responded to messages seeking clinical care will be managed by the delegated clinician researcher using data from the REDCap and TextCare™ platforms.

### Process evaluation

A mixed methods process evaluation of the *SmilesUp* text messaging intervention will be conducted to determine the fidelity, reach, barriers, and enablers to scale the program. Quantitative analysis of fidelity will be measured by exposure (number of text messages delivered and received) and engagement (participant responsiveness to the text messages) [48]. Reach will be measured by the diversity of the participants, and locations using the baseline demographic variables of the study.

To complement the quantitative data; qualitative data collection and analysis will also be conducted to determine the barriers, enablers, and opportunities to scale future text message programs. A convenience and purposive sample reflective of the ethnic and cultural diversity of parents that participated in the program. Framework analysis will be used to analyse qualitative data collected through focus groups and semi-structured interviews [32]. Using the snowballing recruitment technique, we will recruit until the saturation of themes is obtained and anticipate this to be around 20 participants. Similarly, to understand the health system barriers, enablers, and opportunities to scale the program, health professionals (clinician/ academic/ public health professional) will also be invited to attend separate focus groups or semi-structured interviews either conducted in person or via telephone or video call. The focus groups and semi-structured interviews will be conducted by a trained researcher. Those attending in person will be reimbursed for their travel and parking expenses.

### Ethical considerations

Ethics approval has been obtained from the Western Sydney Local Health District Human Research Ethics Committee 2022/ETH01920. The study is currently recruiting with data collection completion anticipated for September 2024. Any amendments to the study will be made in consultation with all principal and site investigators and the Western Sydney Local Health District Human Research Ethics Committee who provided the original approval. This includes changes to eligibility criteria, and analyses, or the addition of sites. Our original protocol version titled *SMILESup_protocol_V2_181122* which included the intervention development as a separate phase [31] was approved by the ethics committee on the 1^st^ of December 2022. Our current protocol version titled *SMILESup_protocol_V5_260923*, focused on the addition of two new study sites and was approved by the ethics committee on the 10^th^ of October 2023. This trial has also been prospectively registered with the Australia and New Zealand Clinical Trials Registry. The ANZCTR is an online register of clinical trials taking place in Australia and New Zealand. It is one of the first three trial registries to be recognised by the World Health Organization International Clinical Trials Registry Platform (WHO ICTRP) as a Primary Registry. Our registration number for the study is ACTRN12623000325606.

## Discussion

The widespread use of mobile phones presents an opportunity for dental researchers, health system managers, policymakers, and health professionals to consider mHealth programs that directly support parents in managing modifiable behavioural risk factors related to ECC [14]. Current preventive disease management approaches for ECC focus on behavioural-focused interventions in clinical settings like Motivational Interviewing (MI) which has shown mixed results and is resource intensive [49]. These MI methods also rely on additional face-to-face interactions, which may not be feasible for time-constrained parents seeking convenient, home-based, nudge-type interventions to establish oral health habits [24].

To address this gap, the *SmilesUp* mobile health intervention has been co-designed to engage parents in managing ECC risk in a semi-personalised way that integrates with their daily lives. This single-blinded RCT protocol outlines the rigorous methods used to test the effectiveness of the intervention with a diverse range of parents in NSW. If successful *SmilesUp* offers an innovative, and cost-effective program to support behaviour alongside the family’s journey of receiving clinical care. By choosing to utilise low-cost and simple text messages, our mode of delivery seeks to minimise the digital health literacy divide [50]. Our mHealth program is not prohibitive to parents who cannot afford to download apps that use a lot of data. It is also not reliant on poor internet connectivity which may affect parents in rural and regional areas. Therefore, text messages were chosen as a modality that we could use to provide the *SmilesUp* text messages equitably and at scale.

We anticipate that the program will improve oral health behaviours positively and increase the parent’s oral health self-efficacy in the medium-short term. Through a comprehensive process evaluation reaching a broad range of families accessing public dental care including DGA across metro, rural and regional local health districts, it also seeks to understand the implementation process by understanding what works, for whom and in which context.

Future research designs that are interoperable with electronic oral health record systems will enable the measurement of longer-term ECC oral health clinical outcomes from these programs, including decayed missing and filled teeth (dmft/DMFT) and dental general anaesthetic service utilisation patterns for the families who have received the text message program. Equally this program may be integrated with other successful digital public health programs for parents related to lifestyle habits for childhood development [51,52] Finally, our outlined methods in this protocol paper and learnings from our process evaluation could inform similar development, adaption and translation of mHealth programs for patients of different cultural backgrounds, ages with other oral or general health conditions.

### Dissemination

Study results will be disseminated via peer-reviewed publications, reports to health service managers, and national and international conferences presentations.

## Conclusion

The SMILESup study will provide parents of children diagnosed with ECC support to improve oral health behaviours like tooth brushing, reducing sugary foods and drinks and maintaining oral health promoting bedtime routines. Our robust evaluation of oral health behaviours and oral health self-efficacy outcomes will provide the initial evidence to expand the scale of the program. Our process evaluation with parents and clinicians across a broad range of public dental settings will also inform nuanced implementation strategies and ways to sustainably embed our program throughout public dental health settings in Australia.

## Supporting information

S1 Checklist Supplementary document 1 SPIRIT CHECKLIST. (DOCX)
